# *Petasites japonicus* Leaves Alleviate Depression in Dextran Sulfate Sodium-Induced Colitis Mice Through the BDNF/TrkB Pathway and Modulation of Inflammation

**DOI:** 10.3390/ijms27073274

**Published:** 2026-04-04

**Authors:** Hwa Rang Na, Hyo Lim Lee, Hye Ji Choi, Yu Mi Heo, Yeong Hyeon Ju, Hyun-Jin Kim, Ho Jin Heo

**Affiliations:** Division of Applied Life Science (BK21), Institute of Agriculture and Life Science, Gyeongsang National University, Jinju 52828, Republic of Korea; hrna@gnu.ac.kr (H.R.N.); gyfla059@gnu.ac.kr (H.L.L.); hjchoi0820@gnu.ac.kr (H.J.C.); yumi@gnu.ac.kr (Y.M.H.); ju8172001@gnu.ac.kr (Y.H.J.); hyunjkim@gnu.ac.kr (H.-J.K.)

**Keywords:** *Petasites japonicus*, depression, gut–brain axis, TLR4, BDNF, colitis

## Abstract

Inflammatory bowel disease (IBD) is a chronic gastrointestinal disorder with a high incidence of anxiety and depression. However, the underlying mechanisms of these symptoms remain to be fully elucidated. This study investigated the effects and mechanisms of a 20% ethanolic extract of *Petasites japonicus* leaves (EPJ) on dextran sulfate sodium (DSS)-induced colitis and depression-like behaviors. The physiological compounds identified in the EPJ were citric acid, chlorogenic acid, caffeic acid, fukinolic acid, 3,5-dicaffeoylquinic acid, quercetin 3-O-β-D-glucose-6″-acetate, 4,5-dicaffeoylquinic acid, kaempferol-3-O-(6″-acetyl)-β-glucopyranoside, and pedunculoside. EPJ significantly alleviated DSS-induced colitis, as evidenced by improvements in body weight loss (87.41% vs. 76.02% in the DSS group), colon length (5.75 vs. 4.34 cm), intestinal permeability (52.80 vs. 163.01 μg/mL), and myeloperoxidase (MPO) activity (0.24 vs. 0.67 U/mg) (*p* < 0.05). Histological analysis further confirmed recovery of goblet cells and attenuation of muscle layer thickening. EPJ also reversed DSS-induced gut microbiota dysbiosis and contributed to the restoration of microbial homeostasis. Behavioral assessments showed that EPJ effectively ameliorated depression-like behaviors. EPJ improved antioxidant systems in colon and brain tissues by modulating malondialdehyde (MDA) levels and reduced glutathione (GSH) and superoxide dismutase (SOD) activity. EPJ further upregulated tight junction protein expression and suppressed TLR4/NF-κB inflammatory pathway activation in both colon and brain tissues. Moreover, EPJ modulated serum stress-related hormones, normalized hypothalamic–pituitary–adrenal (HPA) axis dysregulation, regulated the BDNF/TrkB signaling pathway, and modulated tryptophan–kynurenine metabolism. Collectively, these findings suggest that EPJ exerts protective effects against DSS-induced colitis and depression-like behaviors.

## 1. Introduction

Inflammatory bowel disease (IBD) encompasses chronic inflammatory pathologies of the gastrointestinal tract, exemplified by Crohn’s disease and ulcerative colitis (UC) [1]. Although the precise etiology of IBD remains unclear, it is known to be associated with genetic susceptibility, microbial dysbiosis, and immune dysfunction [1]. Additionally, IBD manifests with symptoms including abdominal pain, diarrhea, intestinal bleeding, and malnutrition, and is often accompanied by various extraintestinal complications [2]. A noteworthy fact is that the incidence of psychiatric disorders, including anxiety and depression, is reported to be approximately three times higher in IBD patients than in the general population [2,3]. Depression-like behaviors have also been observed in mice with dextran sulfate sodium (DSS)-induced colitis, which are thought to be mediated by the complex interplay among inflammatory responses, gut microbiota alterations, and neuroendocrine dysregulation [4,5]. Therefore, this close association between chronic intestinal inflammation and psychiatric disorders suggests the need for therapeutic approaches based on the bidirectional communication system of the gut–brain axis (GBA) [6].

The intestinal barrier is primarily composed of intestinal epithelial cells (IECs) and tight junction (TJ) proteins that collectively maintain structural integrity and intestinal homeostasis [7]. In UC, however, disruption of the intestinal barrier increases intestinal permeability, allowing pro-inflammatory cytokines, bacteria, and harmful metabolites to enter the systemic circulation and induce systemic inflammation [8,9]. Circulating inflammatory cytokines enter the central nervous system (CNS) by increasing the permeability of the blood–brain barrier (BBB) and activate microglia [10,11]. Activated microglia produce iNOS and reactive oxygen species (ROS), leading to neuroinflammation and neuronal cell death [12,13]. Additionally, pro-inflammatory cytokines overactivate the hypothalamic–pituitary–adrenal (HPA) axis, increasing the secretion of stress hormones such as cortisol [14]. Chronic exposure to cortisol has been shown to suppress the BDNF/TrkB pathway, which impairs neurogenesis and synaptic plasticity [15,16]. Thus, intestinal inflammation-induced increased permeability may contribute to the onset of depression by promoting neuroinflammation, HPA axis dysregulation, and impaired neuroplasticity.

Increased intestinal inflammation and permeability can disrupt the intestinal microenvironment, leading to dysbiosis [7,17,18]. Intestinal dysbiosis is generally characterized by a decrease in beneficial commensal bacteria such as *Lactobacillus* and *Bifidobacterium* and a corresponding increase in opportunistic or pathogenic bacteria, including *Escherichia*-*Shigella*, *Desulfovibrio*, and *Bacteroides* [8,19,20]. Consequently, there is an altered production of microbial metabolites, such as short-chain fatty acids and tryptophan (TRP) metabolites, which can reach or interact with the CNS through the lymphatic and circulatory systems to influence brain function and emotional regulation [21,22]. In addition, the gut microbiota produces neurotransmitters such as dopamine, serotonin, and γ-aminobutyric acid (GABA), which regulate mood and anxiety, and dysbiosis can disrupt this process, thereby contributing to the development of depressive symptoms [22]. Hence, intestinal inflammation and microbial dysbiosis can induce neuroinflammation, HPA axis dysregulation, neurotransmitter imbalance, and altered microbial metabolite production through the GBA to ultimately contribute to depression. Therefore, a potential strategy to improve IBD-associated depression is to regulate both intestinal inflammation and the gut microbiota to restore GBA homeostasis.

*Petasites japonicus*, a perennial herbaceous plant of the Asteraceae family, is widely distributed across East Asian countries, including Korea and Japan [12]. The stems and roots of *P. japonicus* have traditionally been used to treat and prevent migraines, tension-type headaches, and gastrointestinal spasms [23]. *P. japonicus* contains bioactive compounds such as chlorogenic acid, fukinolic acid, ferulic acid, and rutin, which are recognized for their anti-allergic, anti-inflammatory, and antioxidant properties [24,25]. Recent studies have reported that *P. japonicus* leaves extract exhibits neuroprotective and cognitive-enhancing effects [12,24]. However, the contribution of *P. japonicus* leaves to gut health and GBA regulation has not been thoroughly investigated. Our previous in vitro study demonstrated that a 20% ethanolic extract of *P. japonicus* leaves (EPJ) promotes the growth of probiotic strains and improves the viability of intestinal (HT-29) and hippocampal (HT22) cells [25]. Based on previous findings, EPJ may have potential as a functional food ingredient or nutraceutical for the management of IBD-associated depression. Therefore, we conducted this study to evaluate the efficacy of EPJ in ameliorating DSS-induced colitis and depression-like behaviors in mice by modulating the GBA. In this context, it may provide insight into the potential role of EPJ in GBA regulation and its relevance to intestinal inflammation and associated neurobehavioral disorders.

## 2. Results

### 2.1. Identification of the Physiological Compounds

**Figure 1:**
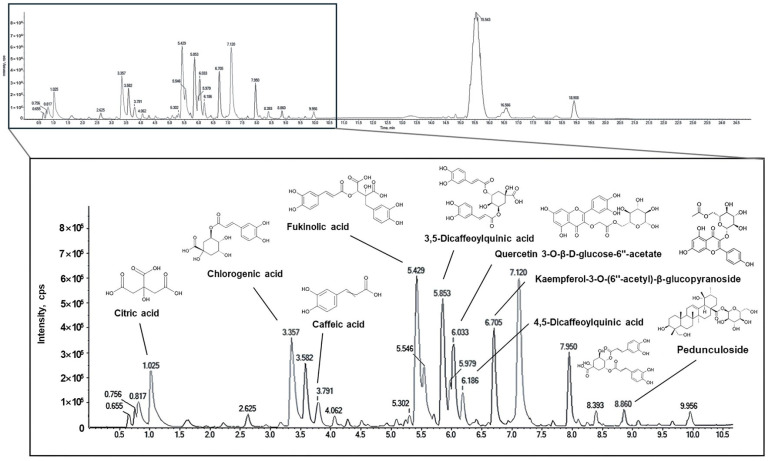
UPLC-Q-TOF-MS/MS chromatogram of EPJ.

The physiological compounds comprising the EPJ were identified using UPLC-Q-TOF-MS/MS (Figure 1 and Table 1). Based on this analysis, the identified compounds included citric acid, chlorogenic acid, caffeic acid, fukinolic acid, 3,5-dicaffeoylquinic acid, quercetin 3-O-β-D-glucose-6″-acetate, 4,5-dicaffeoylquinic acid, kaempferol-3-O-(6″-acetyl)-β-glucopyranoside, and pedunculoside as the major compounds of EPJ. The MS/MS fragmentation patterns of fukinolic acid (*m*/*z* 433 → 271, 253) and kaempferol-3-O-(6″-acetyl)-β-glucopyranoside (*m*/*z* 489 → 284, 255) were consistent with previously reported spectra, confirming their accurate identification, whereas the other compounds were annotated based on library matching [26].

### 2.2. Ameliorative Effect of EPJ on DSS-Induced Colitis Symptoms

**Figure 2:**
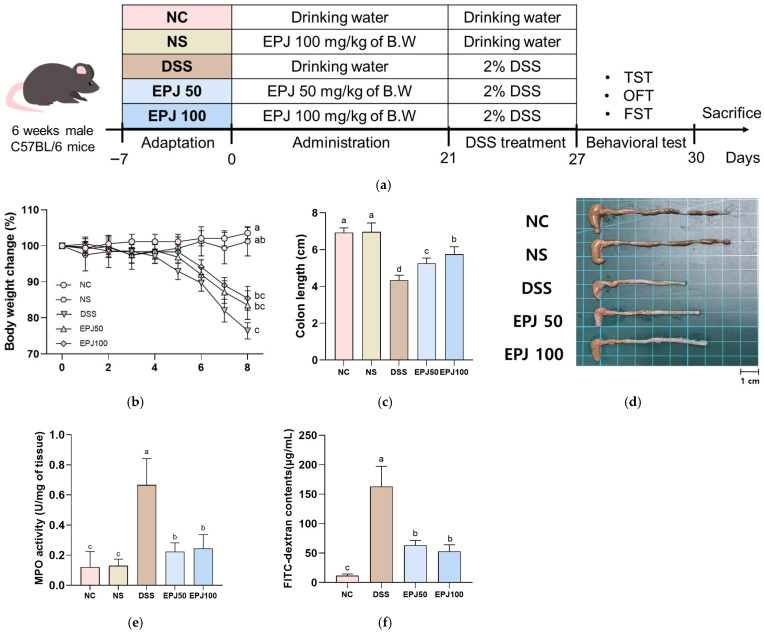
Effect of EPJ on DSS-induced colitis in mice. Schematic illustration of the experimental design and treatment groups (**a**), body weight changes (**b**), colon length (**c**), image of colon tissues (**d**), and MPO activity (**e**) (*n* = 7). FITC-dextran contents (**f**) (*n* = 3). All values are expressed as the mean ± SD. Groups labeled with different lowercase letters differ significantly (*p* < 0.05). Statistical analysis was performed using the Kruskal–Wallis test with Dunn’s post hoc test (**b**), and one-way ANOVA followed by Duncan’s post hoc test (**c**,**e**,**f**). Normal control (NC) group: administered drinking water; normal sample (NS) group: administered EPJ 100 mg/kg of body weight; dextran sulfate sodium (DSS) group: administered DSS and drinking water; EPJ50: administered DSS and EPJ 50 mg/kg of body weight; EPJ100: administered DSS and EPJ 100 mg/kg of body weight.

To examine colitis symptoms in DSS-induced mice, body weight change rate, colon length, intestinal permeability, and MPO activity were measured (Figure 2). The body weight change rate was decreased in the DSS group (76.02%) compared to the NC group (103.48%), while the EPJ 50 and EPJ 100 groups (83.69% and 87.41%, respectively) showed a significant improvement (Figure 2a). The colon length was decreased in the DSS group (4.34 cm) compared to the NC group (6.92 cm), whereas the EPJ 50 and EPJ 100 groups (5.25 cm and 5.75 cm, respectively) exhibited increased colon lengths (Figure 2b,c). MPO activity was increased in the DSS group (0.67 U/mg) compared to the NC group (0.12 U/mg), while the EPJ 50 and EPJ 100 groups (0.22 U/mg and 0.24 U/mg, respectively) showed a significant decrease (Figure 2d). The serum FITC-dextran contents were increased in the DSS group (163.01 μg/mL) compared to the NC group (11.59 μg/mL), while the EPJ 50 and 100 groups (63.18 μg/mL and 52.80 μg/mL, respectively) showed a significant decrease (Figure 2e). The NC and NS groups (102.96%, 6.97 cm, and 0.13 U/mg, respectively) showed no significant differences in body weight change, colon length, and MPO activity (Figure 2a–d).

### 2.3. Effect of EPJ on Histopathological Change in DSS-Induced Colitis

**Figure 3:**
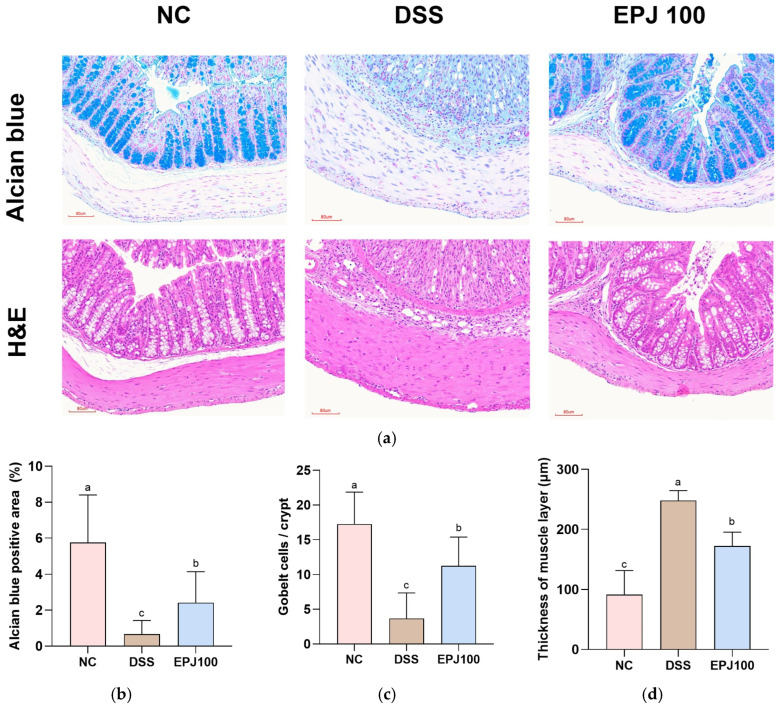
Effect of EPJ on histopathological changes in colon tissue of DSS-induced mice. Representative images of Alcian blue-stained sections and H&E-stained sections (**a**) of colon tissues. Alcian blue positive area (%) (**b**), quantification of goblet cells per crypt (**c**), and thickness of muscle layer (**d**). All values are expressed as the mean ± SD (*n* = 3). Groups labeled with different lowercase letters differ significantly (*p* < 0.05). Statistical analysis was performed by one-way ANOVA with post hoc Duncan’s multiple range test. Normal control (NC) group: administered drinking water; dextran sulfate sodium (DSS) group: administered DSS and drinking water; EPJ100 group: administered DSS and EPJ 100 mg/kg of body weight.

Histopathological evaluation of colon tissues was conducted using Alcian blue and H&E staining (Figure 3). The DSS group exhibited characteristic pathological alterations associated with colitis, including crypt damage, goblet cell depletion, inflammatory cell infiltration, and thickening of the muscularis propria (Figure 3a). The Alcian blue-positive area (%) was significantly reduced in the DSS group (0.67%) compared with the NC group (5.76%), whereas the EPJ 100 group (2.42%) showed a marked improvement (Figure 3b). The number of goblet cells per crypt was markedly decreased in the DSS group (3.67) compared with the NC group (17.27), whereas the EPJ 100 group (11.27) showed a significant recovery (Figure 3c). Furthermore, the muscle layer thickness was significantly increased in the DSS group (248.00 μm) compared with the NC group (91.69 μm), but was notably reduced in the EPJ 100 group (172.56 μm) (Figure 3d).

### 2.4. Effect of EPJ on Gut Microbiome Composition in DSS-Induced Colitis

**Figure 4:**
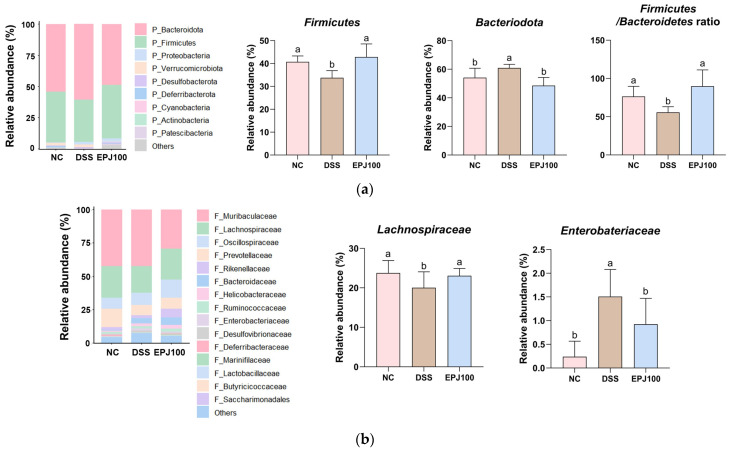

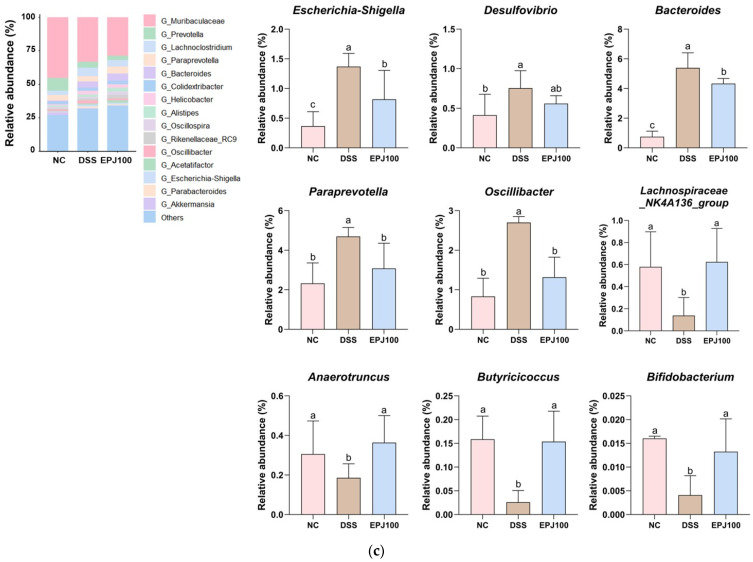
Effect of EPJ on gut microbiome composition of DSS-induced colitis mice. Relative abundance (%) at the phylum (**a**), family (**b**), and genus (**c**) level of each group. All values are expressed as the mean ± SD (*n* = 3). Groups labeled with different lowercase letters differ significantly (*p* < 0.05). Statistical analysis was performed by one-way ANOVA with post hoc Duncan’s multiple range test. Normal control (NC) group: administered drinking water; dextran sulfate sodium (DSS) group: administered DSS and drinking water; EPJ100 group: administered DSS and EPJ 100 mg/kg of body weight.

To evaluate the regulatory effects of EPJ on the gut microbiota, changes in microbial community composition were assessed, and the relationships between microbial taxa and treatment groups were analyzed (Figure 4).

At the phylum level, the relative abundance of *Firmicutes* and the *Firmicutes*/*Bacteroidota* ratio were significantly decreased in the DSS group (33.79% and 55.66%, respectively) compared to the NC group (40.73% and 76.39%, respectively). In contrast, the EPJ 100 group (42.87% and 89.86%, respectively) showed significant improvement. In addition, the relative abundance of *Bacteroidota* was significantly higher in the DSS group (60.92%) than in the NC group (54.07%), whereas the EPJ 100 group (48.59%) showed a significant decrease (Figure 4a).

At the family level, the relative abundance of *Lachnospiraceae* was lower in the DSS group (20.05%) than in the NC group (23.77%), whereas the EPJ 100 group (23.05%) showed significant restoration. However, *Enterobacteriaceae* were significantly increased in the DSS group (1.51%) compared to the NC group (0.24%), whereas the EPJ 100 group (0.60%) showed a significant decrease (Figure 4b).

At the genus level, *Escherichia*-*Shigella*, *Desulfovibrio*, *Bacteroides*, *Paraprevotella*, and *Oscillibacter* were upregulated in the DSS group (1.37%, 0.75%, 5.39%, 4.69%, and 2.69%) compared to the NC group (0.37%, 0.41%, 0.75%, 2.32%, and 0.83%). In comparison, the EPJ 100 group (0.82%, 0.56%, 4.33%, 3.08%, and 1.32%) was downregulated. In contrast, *Lachnospiraceae*_NK4A136, *Anaerotruncus*, *Butyricicoccus*, and *Bifidobacterium* were significantly downregulated in the DSS group (0.14%, 0.19%, 0.03% and 0.00%) compared to the NC group (0.58%, 0.31%, 0.16%, and 0.02%), while the EPJ 100 group (0.62%, 0.36%, 0.15%, and 0.01%) was significantly upregulated (Figure 4c).

### 2.5. Effect of EPJ on Depression-like Behaviors in DSS-Induced Mice

**Figure 5:**
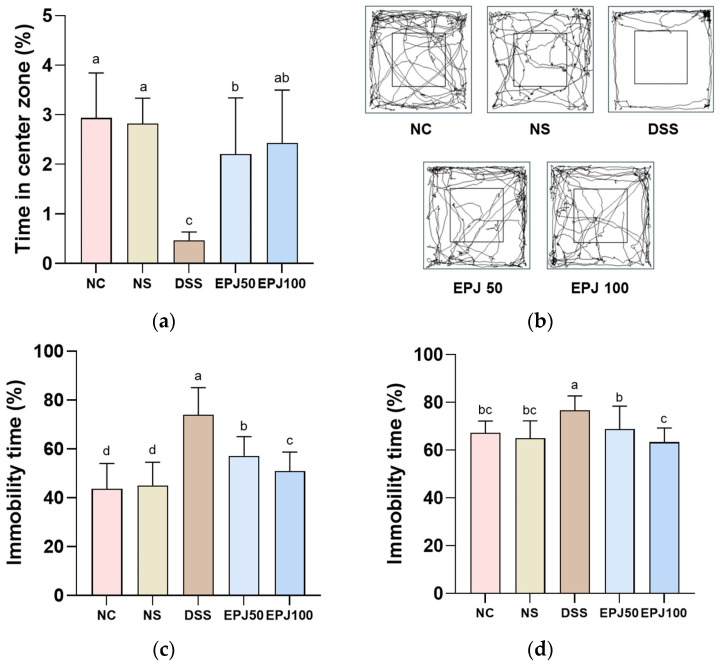
Effect of EPJ on depression-related behaviors of DSS-induced colitis mice. Time in the center zone in OFT (**a**), locomotor tracking image in OFT (**b**), immobility time in TST (**c**), and FST (**d**). All values are expressed as the mean ± SD (*n* = 7). Groups labeled with different lowercase letters differ significantly (*p* < 0.05). Statistical analysis was performed by one-way ANOVA with post hoc Duncan’s multiple range test. Normal control (NC) group: administered drinking water; normal sample (NS) group: administered EPJ 100 mg/kg of body weight; dextran sulfate sodium (DSS) group: administered DSS and drinking water; EPJ50 group: administered DSS and EPJ 50 mg/kg of body weight; EPJ100 group: administered DSS and EPJ 100 mg/kg of body weight.

To evaluate the antidepressant-like effects of EPJ in DSS-induced mice, behavioral tests were conducted (Figure 5).

In the OFT, the time spent in the center zone was significantly reduced in the DSS group (0.47%), compared to the NC group (2.94%), whereas the EPJ 50 and EPJ 100 groups (2.20% and 2.43%, respectively) showed a significant increase (Figure 5a). In the OFT path tracing results, the DSS group predominantly stayed in the peripheral zone compared to the NC and NS groups. In contrast, the EPJ 50 and EPJ 100 groups spent more time in the center zone compared to the DSS group (Figure 5b).

In the TST, the immobility time was significantly increased in the DSS group (74.05%) compared to the NC group (43.71%). In contrast, the EPJ 50 and EPJ 100 groups (57.13% and 50.98%, respectively) showed significantly decreased immobility time compared to the DSS group (Figure 5c).

In the FST, the immobility time was significantly increased in the DSS group (76.76%) compared to the NC group (67.18%). However, the EPJ 50 and EPJ 100 groups (68.87% and 63.40%, respectively) showed significantly decreased immobility time compared to the DSS group (Figure 5d). The NC group and NS group (2.82%, 45.04%, and 65.06%, respectively) showed no significant differences in the parameters in the OFT, TST, and FST (Figure 5a–d).

### 2.6. Protective Effect of EPJ Against DSS-Induced Antioxidant System Dysfunction

**Figure 6:**
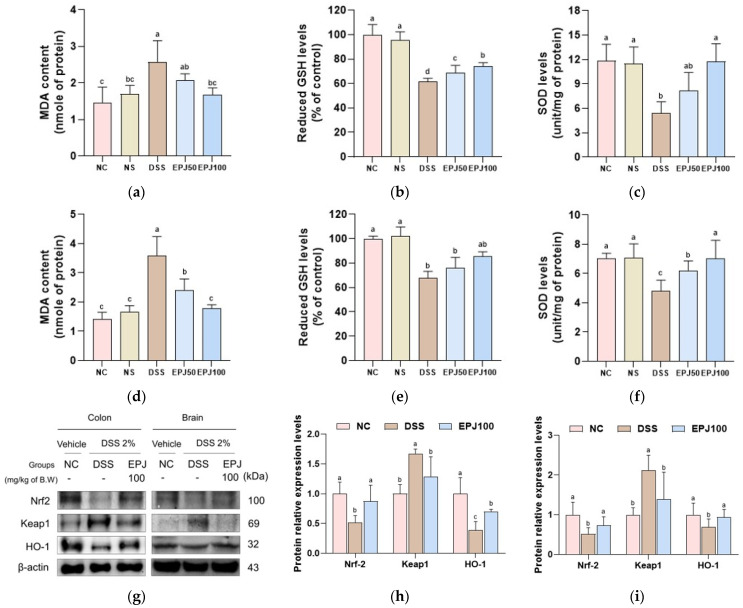
Effect of EPJ on the antioxidant system in DSS-induced colitis mice. MDA contents, reduced GSH levels, and SOD levels in colon (**a**–**c**) and brain (**d**–**f**) tissues (*n* = 7). Representative Western blotting images (**g**), protein expression level of Nrf2, Keap1, and HO-1 in colon (**h**) and brain (**i**) tissues (*n* = 3). All values are expressed as the mean ± SD. Groups labeled with different lowercase letters differ significantly (*p* < 0.05). Statistical analysis was performed using the Kruskal–Wallis test with Dunn’s post hoc test (**a**,**c**,**e**), and one-way ANOVA followed by Duncan’s post hoc test (**b**,**d**,**f**,**h**,**i**). Normal control (NC) group: administered drinking water; normal sample (NS) group: administered EPJ 100 mg/kg of body weight; dextran sulfate sodium (DSS) group: administered DSS and drinking water; EPJ50 group: administered DSS and EPJ 50 mg/kg of body weight; EPJ100 group: administered DSS and EPJ 100 mg/kg of body weight.

The protective effects of EPJ against DSS-induced dysfunction of the antioxidant system were evaluated in colon and brain tissues (Figure 6).

MDA contents in the colon and brain were markedly increased in the DSS group (2.58 and 3.59 nmol/mg protein, respectively) compared with the NC group (1.45 and 1.42 nmol/mg protein, respectively). However, the EPJ 50 and EPJ 100 groups showed significantly decreased MDA contents in the colon and brain (2.07 and 1.68 nmol/mg protein; 2.40 and 1.78 nmol/mg protein, respectively) (Figure 6a,d).

Reduced GSH levels in the colon and brain were significantly decreased in the DSS group (61.53% and 67.94% of control, respectively) compared with the NC group (100% of control in both tissues). Conversely, the EPJ 50 and EPJ 100 groups showed increased GSH levels in the colon and brain (68.56% and 74.13% of control; 76.30% and 85.81% of control, respectively) (Figure 6b,e).

SOD levels in the colon and brain were decreased in the DSS group (5.44 U/mg protein and 4.81 U/mg protein, respectively) compared with those in the NC group (11.84 U/mg protein and 7.05 U/mg protein, respectively). In contrast, the EPJ 50 and EPJ 100 groups showed a dose-dependent increase in SOD levels in the colon and brain (8.15 U/mg protein and 11.75 U/mg protein; 6.18 and 7.04 U/mg protein, respectively) (Figure 6c,f).

The NC and NS groups showed no significant differences in MDA contents, reduced GSH levels, or SOD levels in the colon and brain (1.70 nmol/mg protein, 95.40% of control, and 11.47 U/mg protein; 1.67 nmol/mg protein, 101.96% of control, and 7.10 U/mg protein, respectively) (Figure 6a–f).

Protein expression levels of nuclear factor erythroid 2-related factor 2 (Nrf2) and heme oxygenase-1 (HO-1) in the colon and brain were markedly decreased in the DSS group (0.52 and 0.51; 0.39 and 0.70, respectively) compared with the NC group (1.00 and 1.00, respectively). However, the EPJ 100 group showed significantly increased expression levels of Nrf2 and HO-1 in the colon and brain (0.88 and 0.73; 0.70 and 0.94, respectively) compared with the DSS group. Conversely, protein expression levels of Kelch-like ECH-associated protein 1 (Keap1) in the colon and brain were markedly increased in the DSS group (1.67 and 2.12, respectively) compared with the NC group (1.00 and 1.00, respectively). In contrast, the EPJ 100 group showed significantly decreased Keap1 expression levels in the colon and brain (1.28 and 1.39, respectively) relative to the DSS group (Figure 6g–i).

### 2.7. Protective Effects of EPJ Against DSS-Induced Inflammation and Barrier Dysfunction

**Figure 7:**
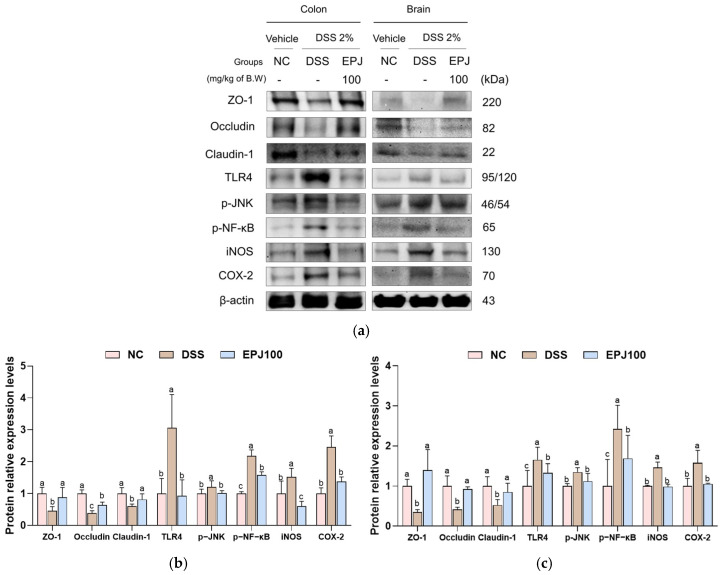
Effect of EPJ on inflammation and barrier dysfunction in DSS-induced colitis mice. Representative Western blotting images (**a**), protein expression level of ZO-1, occludin, claudin-1, TLR4, p-JNK, p-NF-κB, iNOS, and COX-2 in colon (**b**) and brain (**c**) tissues. All values are expressed as the mean ± SD (*n* = 3). Groups labeled with different lowercase letters differ significantly (*p* < 0.05). Statistical analysis was performed by one-way ANOVA with post hoc Duncan’s multiple range test. Normal control (NC) group: administered drinking water; dextran sulfate sodium (DSS) group: administered DSS and drinking water; EPJ100 group: administered DSS and EPJ 100 mg/kg of body weight.

The protective effects of EPJ against DSS-induced TJ disruption and inflammation were evaluated in colon and brain tissues (Figure 7). Protein expression levels of TJ–related proteins, including zonula occludens-1 (ZO-1), occludin, and claudin-1 in the colon and brain were markedly decreased in the DSS group (0.46, 0.39, and 0.60; 0.35, 0.42, and 0.53, respectively) compared with the NC group (1.00). However, the EPJ 100 group showed significantly increased expression levels of these proteins in the colon and brain (0.88, 0.69, and 0.82; 1.40, 0.92, and 0.85, respectively) compared with the DSS group. In parallel, DSS administration markedly upregulated the expression of inflammatory signaling proteins, including TLR4, phosphorylated c-Jun N-terminal kinase (p-JNK), phosphorylated-NF-κB (p-NF-κB), iNOS, and cyclooxygenase-2 (COX-2) in the colon and brain were markedly upregulated in the DSS group (2.25, 1.21, 2.18, 1.46, and 2.45; 1.65, 1.35, 2.42, 1.46, and 1.58, respectively) compared with the NC group (1.00 in both tissues). However, the EPJ 100 group showed significantly decreased expression levels of these proteins in the colon and brain (1.14, 0.87, 1.59, 1.06, and 1.37; 1.33, 1.12, 1.69, 0.99, and 1.06, respectively) compared with the DSS group (Figure 7a–c).

### 2.8. Regulatory Effect of EPJ on DSS-Induced HPA Axis Dysregulation and Synaptic Plasticity Impairment

**Figure 8:**
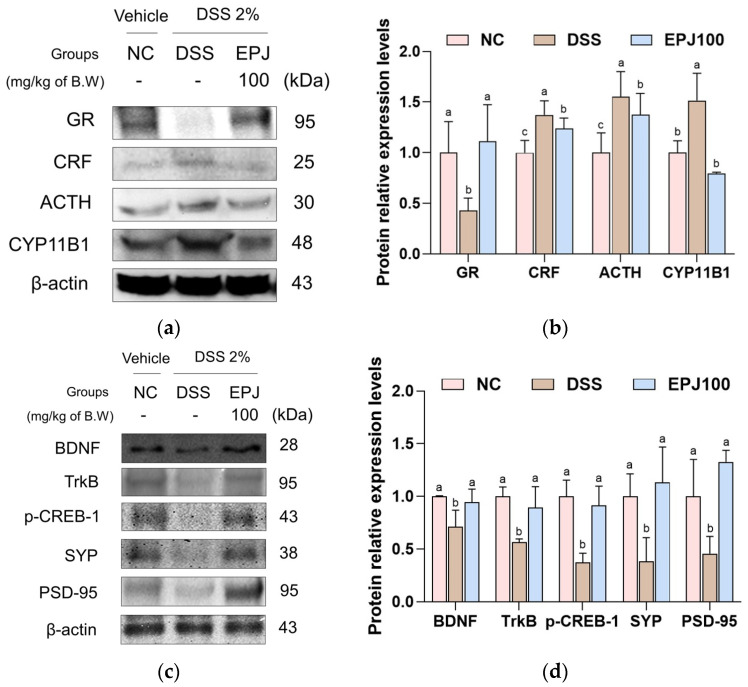
Effect of EPJ on HPA axis and synaptic plasticity in DSS-induced colitis mice. Representative Western blotting images (**a**,**c**) and protein expression level of GR, CRF, ACTH, and CYP11B1 (**b**); and BDNF, TrkB, p-CREB-1, SYP, and PSD-95 (**d**) in brain tissues. All values are expressed as the mean ± SD (*n* = 3). Groups labeled with different lowercase letters differ significantly (*p* < 0.05). Statistical analysis was performed by one-way ANOVA with post hoc Duncan’s multiple range test. Normal control (NC) group: administered drinking water; dextran sulfate sodium (DSS) group: administered DSS and drinking water; EPJ100 group: administered DSS and EPJ 100 mg/kg of body weight.

The regulatory effects of EPJ on DSS-induced HPA axis dysregulation and synaptic plasticity impairment were evaluated in brain tissues (Figure 8). Protein expression levels of glucocorticoid receptor (GR) and synaptic plasticity–related proteins, including BDNF, TrkB, phosphorylated cAMP response element-binding protein 1 (p-CREB-1), synaptophysin (SYP), and postsynaptic density protein-95 (PSD-95), were markedly downregulated in the DSS group (0.63, 0.71, 0.56, 0.37, 0.38, and 0.65, respectively) compared with the NC group (1.00). In contrast, EPJ100 treatment significantly restored the expression of these proteins in brain tissues (1.18, 0.94, 0.90, 0.92, 1.13, and 0.86, respectively). Conversely, the DSS group markedly upregulated the expression of stress-related HPA axis proteins, including corticotropin-releasing factor (CRF), adrenocorticotropic hormone (ACTH), and cytochrome P450 11B1 (CYP11B1) (1.37, 1.55, and 1.58, respectively). EPJ100 treatment (1.24, 1.38, and 0.80, respectively) significantly attenuated the DSS-induced upregulation of these proteins (Figure 8a–d).

### 2.9. Effect of EPJ on DSS-Induced Changes in Hormones and Metabolites

**Figure 9:**
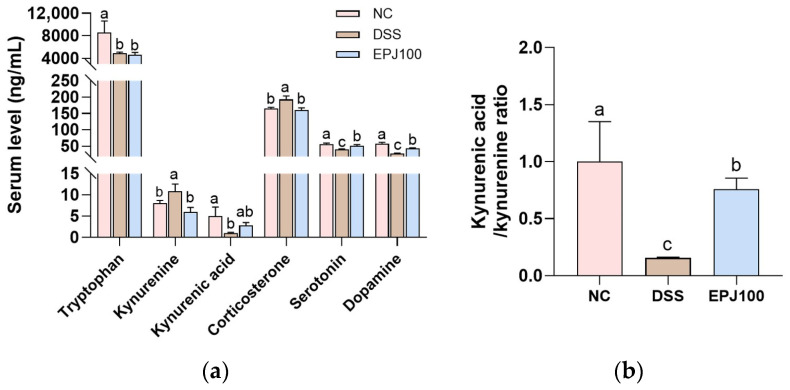

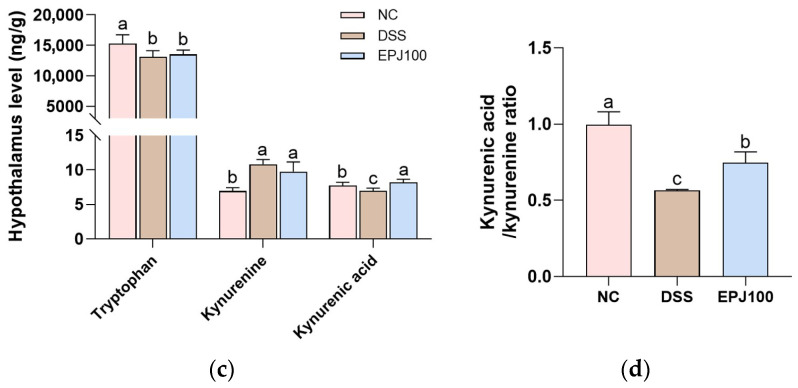
Effects of EPJ on tryptophan metabolites and related hormones in serum (**a**,**b**) and hypothalamus (**c**,**d**) of DSS-induced colitis mice. All values are expressed as the mean ± SD (*n* = 5). Groups labeled with different lowercase letters differ significantly (*p* < 0.05). Statistical analysis was performed by one-way ANOVA with post hoc Duncan’s multiple range test. Normal control (NC) group: administered drinking water; dextran sulfate sodium (DSS) group: administered DSS and drinking water; EPJ100 group: administered DSS and EPJ 100 mg/kg of body weight.

To examine the effect of EPJ on DSS-induced changes in hormones and metabolites, the levels of relevant biomarkers were measured in mouse serum and hypothalamic tissues (Figure 9). The serotonin and dopamine concentrations in the DSS group were markedly decreased (42.70 ng/mL and 27.69 ng/mL, respectively) compared with the NC group (58.05 ng/mL and 57.40 ng/mL, respectively). In contrast, the EPJ group showed restored levels (52.26 ng/mL and 42.49 ng/mL, respectively). In mouse serum, corticosterone levels were significantly increased in the DSS group (193.33 ng/mL) compared with the NC group (165.00 ng/mL). The EPJ group showed a reduction in corticosterone (160.00 ng/mL). TRP levels in serum and hypothalamic tissues did not differ significantly between the DSS group (3665.54 ng/mL and 13,097.15 ng/g) and the EPJ group (4616.89 ng/mL and 13,520.05 ng/g), although both were decreased compared with the NC group (8571.47 ng/mL and 15,280.30 ng/g). Kynurenine (KYN) concentrations in the DSS group were increased in serum (10.77 ng/mL) and hypothalamus (10.83 ng/g) compared with the NC group (8.02 ng/mL and 6.93 ng/g). The EPJ group showed decreased serum KYN (5.81 ng/mL), while hypothalamic levels (9.74 ng/g) remained unchanged. Kynurenic acid (KYNA) levels in the DSS group were decreased in serum (1.02 ng/mL) and hypothalamus (6.99 ng/g) compared with the NC group (5.02 ng/mL and 7.76 ng/g). The EPJ group showed restored KYNA concentrations (2.80 ng/mL in serum and 8.19 ng/g in hypothalamus) (Figure 9a,c). Similarly, the KYNA/KYN ratio in the DSS group was decreased in serum (0.16) and hypothalamus (0.57) compared to the NC group (1.00 and 1.00), while the EPJ group (0.79 and 0.76) showed an apparent restoration toward control levels (Figure 9b,d).

### 2.10. Correlation Analysis

**Figure 10:**
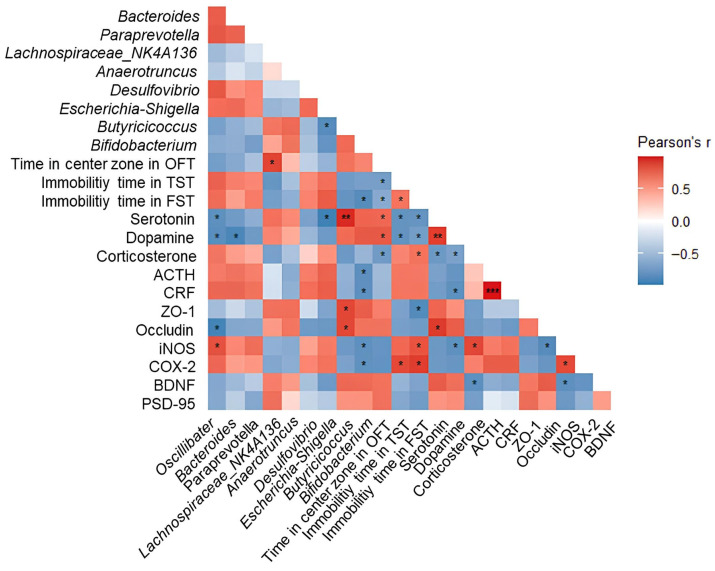
Pearson correlation analysis illustrating the relationships between gut microbial taxa and depression-related behavioral outcomes, circulating hormones, and brain-associated biomarkers. Correlation coefficients are visualized using a color scale, where warmer tones indicate positive associations and cooler tones indicate negative associations. Statistical significance is indicated by asterisks (* *p* < 0.05, ** *p* < 0.01, *** *p* < 0.001) after adjustment for multiple testing using the Benjamini–Hochberg false discovery rate (BH–FDR) method.

To investigate the association between gut microbiota composition and behavioral, hormonal, and depression-related biomarkers in DSS-induced colitis mice, we performed Pearson correlation analyses (Figure 10). Putatively beneficial bacteria such as *Lachnospiraceae*_NK4A136, *Anaerotruncus*, *Butyricicoccus*, and *Bifidobacterium* showed positive correlations with residence time in the central zone of the OFT, serotonin, dopamine, ZO-1, occludin, BDNF, and PSD-95. In contrast, potentially pathogenic bacteria such as *Escherichia*-*Shigella*, *Desulfovibrio*, *Bacteroides*, *Paraprevotella*, and *Oscillibacter* showed negative correlations with these parameters, whereas immobility time of TST and FST showed positive correlations with corticosterone, ACTH, CRF, iNOS, and COX-2.

## 3. Discussion

Although IBD is an idiopathic condition that affects the gastrointestinal tract, studies in both humans and animals demonstrate a close association of inflammatory responses of the intestine with mental disturbances [10]. Therefore, IBD may be effectively treated by a bidirectional therapeutic approach that essentially targets the GBA [6]. Based on this concept, the present research assessed the efficacy of an EPJ as a natural bioactive agent for mitigating DSS-induced colitis and related depression-like symptoms through regulation of the GBA.

In this study, UPLC-Q-TOF-MS/MS analysis was performed to identify the physiologically active compounds present in EPJ (Figure 1 and Table 1). Our findings were consistent with those of previous reports that have identified various polyphenolic compounds, such as chlorogenic acid, fukinolic acid, 3,5-dicaffeoylquinic acid, and 4,5-dicaffeoylquinic acid, in *P. japonicus* extracts [23,24]. Fukinolic acid is a phenolic compound first isolated from *P. japonicus* and is characteristically abundant in this plant, with reported antioxidant and anti-inflammatory activities [23,27]. Chlorogenic acid, a constituent of EPJ, has been shown to attenuate intestinal inflammation in DSS-induced colitis mice by downregulating proinflammatory mediators, such as tumor necrosis factor (TNF)-α and interleukin (IL)-1β, and upregulating TJ proteins, including ZO-1 and occludin [28]. Previous studies have also reported that extracts of *P. japonicus* leaves exert neuroprotective effects by preserving neuronal and synaptic integrity in an Aβ-induced Alzheimer’s disease mouse model [24]. These results suggest that *P. japonicus* can mitigate colitis-associated depression. Therefore, this study aimed to evaluate whether EPJ could alleviate DSS-induced depressive-like behaviors in mice.

The DSS-induced UC model exhibits clinical symptoms and histopathological changes similar to those observed in mice and humans, and is widely used in research on colonic inflammation [29]. DSS administration induces UC symptoms such as weight loss, bloody stools, colonic shortening, and epithelial barrier disruption [20,30]. Neutrophil accumulation is a prominent pathological feature, and colonic MPO activity serves as an indicator of this inflammatory response [31]. In this study, EPJ administration significantly alleviated DSS-induced alterations in body weight, colon length, intestinal barrier integrity, and MPO activity (Figure 2). These physiological and inflammatory changes are accompanied by the typical pathological features of UC, including epithelial barrier disruption and histological damage [32]. Histopathological damage in DSS-induced mice closely resembles clinicopathological features observed in humans, including loss of goblet cells, distortion of the intestinal glandular architecture, and submucosal edema [33]. Goblet cells play a key defensive role in the intestinal mucosa by producing mucus, and their loss compromises the mucosal barrier, increasing susceptibility to colitis [33]. In the present study, EPJ treatment markedly improved the DSS-induced histopathological abnormalities in colonic tissue (Figure 3). A previous report demonstrated that chlorogenic acid, a principal constituent of *P. japonicus*, mitigated DSS-induced reductions in body weight and restored intestinal barrier function in mice [28]. Furthermore, caffeic acid, a phenolic compound found in *P. japonicus*, significantly attenuated colonic histopathological damage, as assessed by H&E staining, in DSS-induced colitis mice [34]. These findings indicate that EPJ, containing bioactive compounds from *P. japonicus*, has the potential to improve IBD symptoms and histopathological damage.

IBD patients are more prone to psychiatric disorders such as anxiety, depression, and bipolar disorder, with the prevalence of depression in adults with IBD reported to be approximately 21–25.2% [3]. Previous studies have reported that DSS-induced colitis in mice is associated with depression- and anxiety-like behaviors, which are commonly assessed using the OFT, TST, and FST [4,5,6]. In the present study, we demonstrate that EPJ alleviates depression-related behavioral abnormalities in DSS-induced colitis (Figure 5). It is known that depressive symptoms in DSS-induced colitis mice are partially regulated by the GBA [3]. Subsequently, to clarify the underlying mechanisms responsible for the antidepressant effects of EPJ, further experiments were conducted focusing on the GBA in both the colon and brain tissues.

Chronic intestinal inflammation due to IBD induces oxidative stress through excessive production of ROS, which are considered critical mediators in the initiation and development of the disorder [35]. Excess ROS accumulation damages cellular macromolecules and inhibits antioxidant defense systems, such as SOD, leading to increased lipid peroxidation products, such as MDA [7,36]. Nrf2 is a key transcription factor that maintains cellular redox homeostasis by modulating antioxidant enzyme expression and serves as a pivotal regulator of defense against oxidative stress [35,37]. Under normal states, Nrf2 binds to Keap1 in the cytoplasm, but oxidative stress disrupts this binding and promotes its nuclear translocation [35]. In the nucleus, Nrf2 activates the transcription of antioxidant genes, such as NAD(P)H quinone dehydrogenase 1 (NQO1) and HO-1 [37]. Such Nrf2 signaling alleviates inflammatory responses and mucosal damage in IBD through antioxidant action [35]. Recent evidence suggests that oxidative stress arising during the pathogenesis of UC contributes to neurodegeneration in the CNS and is linked to the development of anxiety and depression [28,36]. Due to the brain’s high lipid content and oxygen consumption, it is particularly vulnerable to oxidative stress, and oxidative damage can impair neuronal function [22,37]. Clinical studies have reported that serum MDA levels are elevated in patients with depression compared to controls, while the activities of SOD and GSH-Px are significantly decreased in the prefrontal cortex of rats subjected to chronic unpredictable mild stress-induced depression [36,38]. In this study, EPJ showed an improvement in oxidative stress by regulating the levels of antioxidant biomarkers SOD, reduced GSH, and MDA in colon and brain tissues and activating the Nrf2/HO-1 signaling pathway (Figure 6). A previous report demonstrated that *P. japonicus* extract attenuated Aβ_25–35_-induced ROS accumulation in HT22 cells and promoted the expression of antioxidant enzymes, HO-1 and NQO1 [24]. Furthermore, *P. japonicus* extract reduced lipid peroxidation levels and increased total glutathione and GPx in the liver of an L-glutamate-induced mouse model [39]. Therefore, the antioxidant effect of EPJ is considered to alleviate oxidative stress in both the colon and brain, thereby contributing to the improvement of depression-like behaviors.

The intestinal epithelial barrier is a significant defense barrier that protects the intestine from external harmful factors and is essential for preserving intestinal homeostasis in response to gut microbiota and mucosal immune signals [30]. It is composed of a mucus layer and IECs interconnected by TJ proteins, which collectively maintain barrier integrity and regulate permeability [7,28]. However, oxidative stress weakens TJ integrity and compromises barrier function, thereby increasing intestinal permeability [30,35]. This increased permeability disrupts the intestinal microenvironment, leading to microbial dysbiosis, which in turn promotes the production of lipopolysaccharide (LPS), an endotoxin derived from pathogenic bacteria [7,18,29]. LPS is sensed by TLR4, activating JNK and NF-κB signaling pathways and, consequently promoting the excessive release of TNF-α and IL-1β [5,40]. Moreover, these inflammatory cytokines increase intestinal permeability by suppressing the expression of TJ proteins such as ZO-1, occludin, and claudins, and then pass through the damaged gut barrier into the circulation and induce systemic inflammation [11,29]. Circulating cytokines can subsequently disrupt BBB integrity by altering TJ expression in cerebral endothelial cells, allowing inflammatory mediators to enter the brain and activate neuroinflammatory processes [10]. Consequently, inflammatory cytokines gain access to the brain parenchyma, activate microglia, and influence neuroinflammatory processes [10]. In this study, EPJ improved intestinal and BBB dysfunction by upregulating TJ protein expression. In addition, EPJ significantly downregulated inflammatory mediators, including COX-2 and iNOS, in both the colon and brain, indicating its anti-inflammatory potential (Figure 7). Previous studies have shown that *P. japonicus* extract suppressed the expression of pro-inflammatory cytokines, including TNF-α, IL-1β, and IL-6 in an Aβ oligomer-induced neuroinflammation mouse model [12]. These results suggest that EPJ protects the structural integrity of the intestinal barrier and the BBB by regulating TJ protein expression, and alleviates intestinal and neural inflammation by modulating the TLR4/JNK/NF-κB signaling pathway.

Inflammatory mediators are markedly upregulated in the serum of IBD patients and in the brains of colitic animals, and can interact with the HPA axis, a stress hormone system [10,14]. Imbalances due to neuroinflammation can disrupt the regulation of the HPA axis, and some neurotransmitters, such as norepinephrine, serotonin, and dopamine, can influence the secretion of corticosteroid hormone (CRH) and ACTH by modulating peripheral cytokines via cortisol levels [41]. As a consequence of these processes, dysregulation of the HPA axis is commonly detected in individuals with depression, often manifested by excessive glucocorticoid secretion or elevated ACTH levels [14]. Activation of the HPA axis occurs when the hypothalamus releases CRH under stress conditions, which stimulates the pituitary gland to secrete ACTH [22]. ACTH reaches the adrenal cortex via the circulatory system, inducing cortisol secretion in humans and corticosterone in rodents [10]. At this time, GR plays an important role in negative feedback by mediating cortisol effects, but desensitization of these receptors results in an inappropriate cortisol response, further increasing cortisol [22]. Increased cortisol secretion impairs BDNF signaling, which is crucial for neuronal survival and synaptic maintenance, thereby diminishing synaptic plasticity and exacerbating depressive symptoms [22]. BDNF is essential for maintaining neuronal viability, regulating synaptic plasticity, and modulating emotional responses, and its effects are primarily mediated by activation of the high-affinity receptor TrkB [42,43]. Activated TrkB initiates downstream signaling pathways such as the mitogen-activated protein kinase pathway, forming a positive feedback loop in which phosphorylated CREB promotes BDNF expression [42]. Activation of the BDNF/TrkB pathway may improve neural circuit function by restoring key synaptic proteins PSD-95 and SYP [32]. In this study, EPJ improved the HPA axis and synaptic plasticity-related factors in brain tissue (Figure 8). In addition, it increases the levels of the neurotransmitters dopamine and serotonin in the serum and decreases the levels of the stress hormone corticosterone (Figure 9). In a previous study, supplementation with chlorogenic acid, a bioactive compound from *P. japonicus*, significantly reduced serum corticosterone levels in a mouse model subjected to restraint stress [28]. Furthermore, *P. japonicus* extract significantly improved the expression of synaptophysin and PSD-95 in the CA3 region of the hippocampus of Aβ_25–35_-induced mouse brains [24]. These results suggest that EPJ can improve DSS-induced depression by normalizing HPA axis dysfunction and enhancing synaptic plasticity through modulation of the BDNF/TrkB pathway. In conclusion, these findings suggest that EPJ alleviates depression- and anxiety-like behaviors associated with DSS exposure by modulating the GBA.

Alterations in gut microbiota composition and diversity play a crucial role in the development of UC, and numerous studies have reported that DSS administration markedly disrupts the microbial community structure in colitis-induced mice [8]. Accordingly, dysbiosis of the gut microbiota is regarded as both a hallmark and a contributing factor in the pathogenesis of UC, with patients typically exhibiting an increase in conditionally pathogenic bacteria and a decrease in beneficial microbes compared to healthy individuals [20]. Moreover, the gut microbiota serves as a key mediator of bidirectional signaling between the gastrointestinal tract and the brain, influencing brain regions involved in stress control [41]. It also contributes to the synthesis of neurotransmitters such as serotonin, dopamine, and GABA, which are crucial for mood and anxiety regulation, and have been linked to major depressive disorder [22]. In IBD, microbial imbalance is characterized by an elevated *Bacteroidota*/*Firmicutes* ratio [44]. DSS treatment increases the abundance of LPS-producing bacteria, such as *Bacteroides* and *Escherichia*-*Shigella*, while decreasing probiotics, such as *Butyricicoccus* and *Lachnospiraceae*_NK4A136 [8,17,19]. Similar results were observed in this study, and EPJ administration restored the imbalance in the microbial community (Figure 4). In particular, bacteria commonly associated with depression, such as *Oscillibacter*, *Desulfovibrio*, and *Paraprevotella*, were significantly elevated in the DSS group, whereas EPJ treatment restored levels of *Bifidobacterium*, a probiotic strain known to alleviate depressive symptoms [45,46]. These findings suggest that EPJ may improve depression by partially modulating the gut microbiota altered by DSS-induced colitis. Additionally, this study performed Pearson correlation analysis to examine the correlation between the gut microbiome and key biomarkers and behavioral indicators associated with depression (Figure 10). The results showed that *Lachnospiraceae*_NK4A136, *Anaerotruncus*, *Butyricicoccus*, and *Bifidobacterium* were negatively correlated with depression markers, whereas *Escherichia*-*Shigella*, *Desulfovibrio*, *Bacteroides*, *Paraprevotella*, and *Oscillibacter* were positively correlated. These results suggest that EPJ modulates neurotransmitter interactions and stress-related signaling within the GBA by restoring gut microbiota composition, thereby alleviating depressive-like behaviors.

By accessing the CNS through lymphatic routes and the bloodstream or by interacting with the enteric nervous system, the metabolites of gut microbiota can influence neural function, mood, and behavior [21]. Among them, TRP is an essential amino acid mainly absorbed in the intestine, producing metabolites involved in emotional regulation, inflammatory response, and brain function, and is closely related to depression [21]. Notably, over 95% of systemic serotonin originates from TRP metabolism within enterochromaffin cells of the GI tract, a process in which the gut microbiota plays a crucial regulatory role [47]. However, chronic inflammation due to IBD leads to hyperactivation of Indoleamine 2,3-dioxygenase (IDO) and alters TRP metabolism, shifting serotonin synthesis toward KYN production and reducing serotonin levels, leading to the production of neurotoxic metabolites [41,48,49]. KYN can penetrate the BBB and is further metabolized into KYNA, which has neuroprotective effects, or into neurotoxic metabolites such as 3-hydroxykynurenine and quinolinic acid [41,50]. A higher KYN/KYNA ratio has been reported to increase neurotoxicity, which contributes to the development of depression [49]. In this study, TRP levels were decreased in both the serum and hypothalamus of DSS-induced mice, including those treated with EPJ, likely due to increased TRP catabolism caused by IDO-1 upregulation in response to inflammation [51]. Under physiological conditions, approximately 80% of KYN in the brain is derived from peripheral blood, but during local immune activation in the central nervous system, more than 98% of KYN is synthesized locally in the brain [51]. Given this physiological linkage between peripheral and central KYN metabolism, EPJ administration markedly restored serum KYN levels, whereas no apparent alteration was detected in the hypothalamus (Figure 9). This finding suggests that IDO-1-mediated local KYN production within the hypothalamus may be increased along with HPA axis dysfunction [1,50]. Moreover, EPJ increased KYNA levels in the serum and hypothalamus and contributed to restoring the balance of the TRP metabolic pathway by regulating the KYNA/KYN ratio. Furthermore, EPJ may indirectly promote the conversion of TRP to serotonin by modulating the gut microbiota. *Bifidobacterium*, which was increased in the EPJ group, is known to promote serotonin synthesis by regulating TRP availability [22]. In this regard, chlorogenic acid has been shown in a previous study to increase serotonin levels and improve depressive-like behaviors through IDO inhibition in a mouse restraint stress model [48]. However, in the DSS model of this study, the strong inflammatory response may have limited the regulation of TRP metabolism through the IDO-1 pathway. Nevertheless, EPJ administration significantly increased serotonin levels compared to the DSS-treated group. These findings indicate that EPJ contributed to alleviating DSS-induced depressive-like behavior by normalizing TRP metabolism in colitic mice (Figure 9). Although our findings suggest a regulatory role of EPJ in inflammation-driven TRP metabolism, further studies are warranted to clarify whether EPJ directly modulates this process by regulating IDO-1 expression.

Overall, our study demonstrates that EPJ alleviates depressive-like behaviors in DSS-induced mice. The underlying mechanisms include suppression of oxidative stress and inflammation, upregulation of TJ proteins, restoration of HPA axis balance, enhancement of the BDNF/TrkB pathway, amelioration of dysbiosis, and regulation of TRP metabolism. Our findings suggest that the GBA plays a critical role in the pathophysiology of DSS-induced colitis, as further supported by the correlation analyses conducted in this study. Finally, our results suggest the need for therapeutic approaches that target the GBA-mediated bidirectional system to manage colitis-induced depression. However, several limitations should be acknowledged. In this study, the mechanisms were primarily inferred from biochemical and molecular biological indicators in in vivo experiments, and the individual contributions of the bioactive compounds in EPJ were not clearly distinguished. In addition, this study focused on TRP metabolism, and other metabolite pathways potentially involved in gut–brain communication, including short-chain fatty acids, bile acids, and lipid mediators, were not investigated. Investigating these metabolites would provide deeper insight into the mechanisms underlying GBA modulation. Further metabolite profiling in intestinal tissues may help to better explain the local metabolic alterations associated with DSS-induced colitis and their contribution to GBA modulation. Future studies should focus on identifying the active compounds and exploring broader metabolite profiles to further elucidate the mechanisms of EPJ, thereby supporting its potential as a functional food for IBD-associated depression.

## 4. Materials and Methods

### 4.1. Preparation of EPJ

The *P. japonicus* leaves used in this study were grown in Geochang-gun (Republic of Korea) and purchased as fresh leaves in February 2024 through Naegohyang Food. *P. japonicus* leaves were dried using a freeze dryer (FDU-8612, operon, Gimpo, Republic of Korea) and ground into a powder. 20 g of the powder and 1 L of 20% ethanol were mixed, followed by reflux cooling extraction at 40 °C for 2 h. The extract was filtered, concentrated, and subsequently lyophilized to obtain the EPJ powder.

### 4.2. Physiological Compounds Analysis

Physiological compounds in EPJ were characterized by UPLC using a Nexera XS system (Shimadzu, Kyoto, Japan) interfaced with an X500R Q-TOF-MS/MS (SCIEX, Framingham, MA, USA). The instrumental analyses were performed at the High-Tech Materials Analysis Core Facility of Gyeongsang National University (Jinju, Republic of Korea). Separation was performed on an ACQUITY UPLC BEH C_18_ column (2.1 × 100 mm, 1.7 μm) using distilled water with 0.1% formic acid (solvent A) and acetonitrile with 0.1% formic acid (solvent B). The detector was set to 254 nm, and the flow rate, column temperature, and injection volume were 0.35 mL/min, 40 °C, and 3 μL, respectively. The gradient program was 0–18 min (0–80% B), 18–20 min (80–0% B), and 20–25 min (0% B). Mass detection was performed in negative electrospray ionization (ESI) mode with a ramp collision energy of 20–50 eV, a capillary voltage of 4.5 kV, an ion-source temperature of 500 °C, and a scan range of *m*/*z* 50–1500.

### 4.3. Animal Experiment Design

We conducted the experiments with the approval of the Gyeongsang National University Animal Experiment Ethics Committee (IACUC approval number: GNU-240808-M0158, date of approval: 8 August 2024). The animals were housed under controlled temperature (22 ± 2 °C), humidity (50–55%), and a 12 h/12 h light/dark cycle. After an adaptation period of one week, the mice were divided into five groups as follows: normal control (NC) group (without DSS treatment, drinking water administration); normal sample (NS) group (without DSS treatment, EPJ administration at 100 mg/kg of body weight); DSS group (DSS treatment, drinking water administration); EPJ50 group (DSS treatment, EPJ administration at 50 mg/kg of body weight); and EPJ100 group (DSS treatment, EPJ administration at 100 mg/kg of body weight). Mice were randomly assigned to experimental groups using the RAND() function in Microsoft Excel to generate a randomization sequence. After 3 weeks of oral EPJ administration, all mice except the NC and NS groups were induced to colitis by adding 2% (*w*/*v*) DSS to their drinking water for 6 days. To minimize potential confounding factors, cage positions were regularly rotated throughout the study, and the order of treatments and sample collections was randomized across groups. Each group consisted of 18 mice (*n* = 90), which were pre-assigned to independent experimental analyses prior to the start of the study, including behavioral tests, antioxidant system analysis, and MPO activity (*n* = 7); FITC-dextran permeability assay (*n* = 3); next-generation sequencing (NGS) analysis, histological staining, and Western blot analysis (*n* = 3); and hormonal and metabolite analysis (*n* = 5). Each individual mouse was considered a biological replicate, and all measurements were obtained from independent samples.

### 4.4. Animal Behavioral Tests

#### 4.4.1. OFT

To perform the OFT, a square box (50 cm × 50 cm × 50 cm) with an open top was divided into 16 equal sections. In these sections, four central squares were designated as center zones, and the remaining 12 squares were designated as the peripheral zones. Each mouse was positioned in the peripheral zone, and its locomotor movement was monitored for 5 min with video-tracking software (Smart 3.0, Panlab, Barcelona, Spain). After each mouse finished the experiment, the box was wiped with 75% ethanol to prevent olfactory cues from affecting subsequent animals.

#### 4.4.2. TST

To perform the TST, each mouse was suspended by its tail using adhesive tape placed approximately 1 cm from the tail tip. The mouse was positioned on an iron rod approximately 50 cm above the floor, ensuring it remained 15 cm above the ground. The duration of immobility was monitored for 5 min using video-tracking software (Smart 3.0, Panlab). Immobility time was defined as the duration during which the mouse remained completely motionless while passively hanging.

#### 4.4.3. FST

To perform the FST, each mouse was placed in a transparent cylindrical tank filled with water (25 °C) to a depth of 15 cm. The animals were allowed to swim for 5 min, and the duration of immobility was monitored using video-tracking software (Smart 3.0, Panlab). Immobility was defined as the absence of active movements, with only minimal movements necessary to keep the head above water. The water in the cylinder was replaced after each trial.

### 4.5. FITC-Dextran Permeability Assay

Mice were fasted for 6 h and then transferred to new cages, where they subsequently received FITC-dextran through oral gavage at a dose of 400 mg/kg body weight. After 4 h, blood was collected from the abdominal vein and centrifuged at 13,000× *g* for 10 min at 4 °C to obtain serum. The serum was diluted 1:5 in phosphate-buffered saline (PBS). Fluorescence intensity was measured using a fluorometer (Infinite 200, Tecan Co., Männedorf, Switzerland) at 485 nm excitation and 535 nm emission. The FITC-dextran contents in serum were determined by comparison with a standard curve.

### 4.6. MPO Activity Analysis

Colon tissues were homogenized in a 0.5% hexadecyltrimethylammonium bromide (HTAB) solution in 0.05 M phosphate buffer (pH 6.0) and centrifuged at 10,000× *g* for 20 min at 4 °C to obtain enzyme extracts, as previously described [52]. The reaction mixture contained 0.05 M potassium phosphate buffer, o-dianisidine dihydrochloride, and 0.0005% H_2_O_2_, and the absorbance was measured at 450 nm using an Epoch 2 microplate reader (BioTek Instruments, Winooski, VT, USA).

### 4.7. Histopathological Analysis

Colon tissues were washed with PBS and fixed in 10% formalin solution. The samples were embedded in paraffin and sectioned into approximately 4-μm-thick slices using the Finese ME Microtome (Thermo Fisher Scientific, Waltham, MA, USA). Alcian blue staining was performed to assess mucin-producing goblet cells, and H&E staining was used to evaluate general histopathological changes. The Alcian blue positive area (%) was quantified using ImageJ Fiji software (version 2.0, National Institutes of Health, Bethesda, MD, USA). Goblet cell numbers and muscle layer thickness were measured from five randomly selected areas per tissue, and all image analyses were performed using ImageJ software (version 1.54d, National Institutes of Health).

### 4.8. NGS Analysis

Genomic DNA was isolated from mouse feces, and 16S rRNA sequencing was conducted by Sanigen Inc. (Anyang, Republic of Korea) using the NextSeq 2000 platform (Illumina, San Diego, CA, USA). The V3-V4 hypervariable regions of the bacterial 16S rRNA gene were amplified by polymerase chain reaction (PCR) using region-specific primers. Paired-end sequencing reads (2 × 300 bp) were processed using QIIME2 (version 2023.02). Quality control of raw reads was conducted using FastQC (version 0.11.8), followed by removal of artificial sequences, primer contaminants, and low-quality bases. Chimeric sequences introduced during the PCR step in library preparation were also eliminated to obtain high-quality sequences. The resulting sequences were taxonomically annotated to the genus level using the SILVA 16S rRNA reference database.

### 4.9. Antioxidant System Activity

The MDA content and the levels of SOD and reduced GSH in colon and brain tissues were measured according to a previously reported procedure [52]. MDA content was measured by homogenizing colon and brain tissues in PBS, followed by centrifugation (3000× *g*, 10 min, 4 °C). The supernatants were reacted with phosphoric acid and thiobarbituric acid, incubated at 95 °C for 1 h, and the absorbance was measured at 532 nm. SOD activity was measured by homogenizing tissues, followed by extraction and centrifugation (10,000× *g*, 10 min, 4 °C). The supernatants were analyzed using a commercial kit (Dojindo Laboratories, Kumamoto, Japan), and absorbance was measured at 450 nm. Reduced GSH levels were measured in supernatants obtained from tissues homogenized in phosphate buffer containing EDTA and centrifuged (10,000× *g*, 15 min, 4 °C). The supernatants were mixed with metaphosphoric acid and centrifuged again. The reaction mixture was added to a solution containing Tris-HCl, NaOH, and o-phthaldialdehyde, followed by incubation in the dark for 15 min. Fluorescence was measured at 360 nm excitation and 430 nm emission.

### 4.10. Hormonal Analysis

Serum levels of serotonin, dopamine, and corticosterone were determined using enzyme-linked immunosorbent assay (ELISA) kits (MyBioSource, San Diego, CA, USA) according to the manufacturer’s recommendations. The absorbance at 450 nm was determined using an Epoch 2 microplate reader (BioTek), and serum levels were calculated from the standard calibration curve.

### 4.11. Tryptophan Metabolite Analysis

The hypothalamic tissue was homogenized in 50% methanol, followed by centrifugation at 20,000× *g* for 15 min at 4 °C. The resulting supernatant was collected for subsequent analysis. For serum preparation, each sample was mixed with methanol and shaken at 4 °C for 30 min before being centrifuged at 24,000× *g* for 10 min. The obtained supernatant was concentrated using a speed-vacuum evaporator (NB-503CIR, N-bioteck, Bucheon, Republic of Korea), redissolved in 50% methanol, and then centrifuged. The final supernatants were subjected to UPLC-Q-TOF-MS/MS analysis using an Xevo™ TQ-S micro system (Waters Corp., Milford, MA, USA). Chromatographic separation was achieved on an ACQUITY UPLC BEH C_18_ column (2.1 mm × 100 mm, 1.7 μm; Waters Corp.), with a mobile phase consisting of distilled water and acetonitrile containing 0.1% formic acid. The elution was performed at a flow rate of 0.35 mL/min, with the column maintained at 40 °C and an injection volume of 1 μL. The electrospray ionization (ESI) source was operated in positive ion mode. The specific multiple reaction monitoring (MRM) transitions and parameters for the analytes are summarized in Table S1.

### 4.12. Western Blot Analysis

Western blot analysis was performed according to previously reported procedures [52]. Briefly, samples were lysed in a lysis buffer supplemented with 1% protease inhibitor. The samples with equalized protein concentrations were denatured, separated by sodium dodecyl sulfate–polyacrylamide gel electrophoresis (SDS-PAGE), and transferred onto polyvinylidene fluoride (PVDF) membranes. The membranes were incubated with primary antibodies for over 12 h at 4 °C. After washing, the membranes were incubated with secondary antibodies for 1 h at room temperature, depending on the host of the primary antibody. The luminescent signals of the protein bands were detected with an iBright CL1000 image analyzer (Thermo Fisher Scientific). The primary antibodies information is summarized in Table S2.

### 4.13. Statistical Analysis

All experimental data were expressed as mean ± SD. Statistical analysis was performed using one-way ANOVA followed by Duncan’s multiple range test with SAS software 9.4 (SAS Institute, Cary, NC, USA). When data were not normally distributed, multiple comparisons were further evaluated using the non-parametric Kruskal–Wallis test and Dunn’s post hoc test using GraphPad Prism 10.6.0 (GraphPad Software, Boston, MA, USA). Pearson’s correlation analysis was performed in RStudio 4.5.1. Statistical significance was considered at *p* < 0.05.

## 5. Conclusions

In conclusion, EPJ alleviated DSS-induced colitis and depressive-like behaviors by modulating GBA. EPJ, which contains various phenolic compounds, enhanced the antioxidant defense system via activation of the Nrf2/HO-1 pathway, accompanied by increased GSH and SOD levels and decreased MDA accumulation, thereby reducing oxidative stress. In addition, EPJ suppressed inflammatory responses by regulating the TLR4/NF-κB signaling pathway and preserved intestinal barrier integrity by modulating TJ proteins. This alleviation of neuroinflammation normalized HPA axis dysfunction, leading to activation of the BDNF/TrkB pathway and restoration of neuroplasticity. Furthermore, EPJ restored gut microbial balance and modulated microbiota-derived neurotransmitters and metabolites. In summary, EPJ has the potential as a natural remedy for improving depression associated with IBD by simultaneously alleviating intestinal inflammation and gut microbiota imbalance.

## Figures and Tables

**Table 1:** Identification of physiological compounds in the EPJ by using UPLC-Q-TOF-MS/MS.

	Retention Time (min)	Compound	Parent Ion (*m*/*z*)	Fragment Ion (*m*/*z*)
1	1.03	Citric acid	191	111, 87, 85, 67
2	3.36	Chlorogenic acid	353	191, 161, 85
3	3.80	Caffeic acid	179	135, 133, 107, 89
4	5.42	Fukinolic acid	433	271, 253
5	5.85	3,5-Dicaffeoylquinic acid	515	353, 191, 179, 135, 134
6	6.03	Quercetin 3-O-β-D-glucose-6″-acetate	505	463, 300, 271, 178
7	6.20	4,5-Dicaffeoylquinic acid	515	353, 191, 179, 173, 93
8	6.70	Kaempferol-3-O-(6″-acetyl)-β-glucopyranoside	489	284, 255
9	8.86	Pedunculoside	695	649, 487

## Data Availability

The original contributions presented in this study are included in the article. Further inquiries can be directed to the corresponding author.

## Supplementary Materials

The following supporting information can be downloaded at: https://www.mdpi.com/article/10.3390/ijms27073274/s1.

## Abbreviations

The following abbreviations are used in this manuscript:

UPLC	Ultra-Performance Liquid Chromatography
Q-TOF-MS/MS	Quadrupole Time-of-Flight Tandem Mass Spectrometry
MDA	Malondialdehyde
SOD	Superoxide dismutase
GSH	Glutathione
TLR4	Toll-like receptor 4
NF-κB	Nuclear factor kappa B
BDNF	Brain-derived neurotrophic factor
TrkB	Tropomyosin receptor kinase B
iNOS	Inducible nitric oxide synthase
FITC	Fluorescein isothiocyanate
MPO	Myeloperoxidase
H&E	Hematoxylin and Eosin
SD	Standard deviation
ANOVA	Analysis of variance
