# Supplementary material for: Petasites japonicus Leaves Alleviate Depression in Dextran Sulfate Sodium-Induced Colitis Mice Through the BDNF/TrkB Pathway and Modulation of Inflammation
Source: Int J Mol Sci. 2026 Apr 4;27(7):3274. doi: 10.3390/ijms27073274 (PMC13073347; doi:10.3390/ijms27073274)
Supplement: Supplementary file 1 [file ijms-27-03274-s001.zip › ijms-4182228-supplementary.pdf]

Table S1. Multiple reaction monitoring (MRM) conditions in the positive electrospray ionization (ESI) mode.

Analytes	Retention time (min)	Precursor ion (*m*/*z*)	Product ion (*m*/*z*)	Collision energy (eV)
Tryptophan	2.61	188.13	188.1	40
Kynurenine	2.40	209.26	94.14	20
Kynurenic acid	2.69	190.02	144.11	20

Table S2. List of primary and secondary antibodies information utilized in this study.

Antibody	Catalog	Manufacturer
Nrf2	ab62352	Abcam (Cambridge, UK)
Keap1	sc-514914	Santa Cruz Biotechnology (Dallas, TX, USA)
HO-1	sc-136960	
ZO-1	sc-33725	
Occludin	sc-133256	
Claudin-1	sc-166338	
TLR4	sc-293072	
p-JNK	sc-6254	
p-NF-κB	sc-136548	
iNOS	sc-7271	
COX-2	sc-37681	
GR	sc-12763	
CRF	sc-293187	
ACTH	sc-57018	
CYP11B1	sc-374096	
TrkB	sc-377218	
p-CREB-1	sc-81486	
SYP	sc-17750	
PSD-95	sc-32290	
β-action	sc-69879	
BDNF	#47808	Cell Signaling Tech (Danvers, MA, USA)
